# Poly[diaqua­(μ_4_-3,5-dicarb­oxyl­ato­pyra­zol-1-ido-κ^6^ *N* ^1^,*O* ^5^:*N* ^2^,*O* ^3^:*O* ^3′^:*O* ^5^,*O* ^5′^)lanthanum(III)]

**DOI:** 10.1107/S1600536809020479

**Published:** 2009-06-06

**Authors:** Jun Xia, Jun-Fu Wei

**Affiliations:** a College of Materials Science and Engineering, Tianjin Polytechnic University, Tianjin 300160, People’s Republic of China

## Abstract

In the title coordination polymer, [La(C_5_HN_2_O_4_)(H_2_O)_2_]_*n*_, the lanthanum(III) metal centre is nine-coordinated, with a distorted tricapped trigonal prismatic geometry, by the O atoms of two water mol­ecules and by two N and five O atoms of two *N*,*O*-bidentate, one *O*,*O*′-bidentate and one *O*-mono­dentate 3,5-dicarb­oxyl­ato­pyra­zol-1-ide ligands. The polymeric three-dimensional structure is stabilized by inter­molecular O—H⋯O hydrogen bonds.

## Related literature

For other coordination complexes with pyrazole-3,5-dicarboxylic acid ligands, see: Sakagami *et al.* (1996 ▶); Wang *et al.* (2007 ▶); Yang *et al.* (2004 ▶); King *et al.* (2003 ▶); Pan *et al.* (2000 ▶).
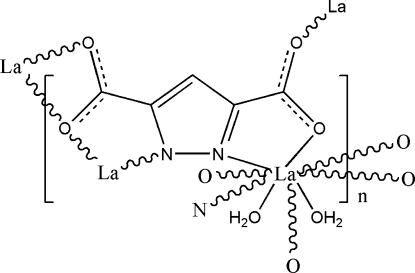


## Experimental

### 

#### Crystal data

[La(C_5_HN_2_O_4_)(H_2_O)_2_]
*M* *_r_* = 328.02
Orthorhombic, 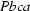

*a* = 12.5712 (8) Å
*b* = 8.4350 (6) Å
*c* = 16.0070 (10) Å
*V* = 1697.35 (19) Å^3^
*Z* = 8
Mo *K*α radiation
μ = 5.04 mm^−1^
*T* = 293 K
0.32 × 0.24 × 0.20 mm

#### Data collection

Bruker APEXII CCD diffractometer
Absorption correction: multi-scan (*SADABS*; Sheldrick, 1996 ▶) *T*_min_ = 0.239, *T*_max_ = 0.365
8554 measured reflections
1496 independent reflections
1316 reflections with *I* > 2σ(*I*)
*R*_int_ = 0.023

#### Refinement

*R*[*F* ^2^ > 2σ(*F* ^2^)] = 0.016
*wR*(*F* ^2^) = 0.041
*S* = 1.06
1496 reflections
128 parameters
H-atom parameters constrained
Δρ_max_ = 0.52 e Å^−3^
Δρ_min_ = −0.50 e Å^−3^

### 

Data collection: *APEX2* (Bruker, 2007 ▶); cell refinement: *SAINT-Plus* (Bruker, 2007 ▶); data reduction: *SAINT-Plus*; program(s) used to solve structure: *SHELXS97* (Sheldrick, 2008 ▶); program(s) used to refine structure: *SHELXL97* (Sheldrick, 2008 ▶); molecular graphics: *SHELXTL* (Sheldrick, 2008 ▶); software used to prepare material for publication: *SHELXTL*.

## Supplementary Material

Crystal structure: contains datablocks I, global. DOI: 10.1107/S1600536809020479/rz2328sup1.cif

Structure factors: contains datablocks I. DOI: 10.1107/S1600536809020479/rz2328Isup2.hkl

Additional supplementary materials:  crystallographic information; 3D view; checkCIF report

## Figures and Tables

**Table 1:** Hydrogen-bond geometry (Å, °)

*D*—H⋯*A*	*D*—H	H⋯*A*	*D*⋯*A*	*D*—H⋯*A*
O5—H5*A*⋯O6	0.85	2.14	2.944 (3)	159
O5—H5*B*⋯O3^i^	0.85	1.82	2.667 (3)	174
O6—H6*A*⋯O1^ii^	0.85	2.20	2.999 (3)	155
O6—H6*B*⋯O1^iii^	0.85	2.12	2.908 (3)	153

Symmetry codes: (i) 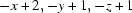; (ii) 
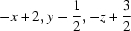
; (iii) 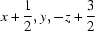.

**Fig. 1.**
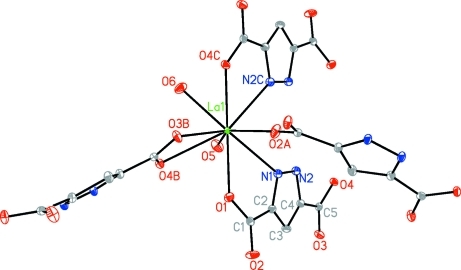
The coordination environment of the lanthanum(III) atom, with the atom-numbering scheme, showing displacement ellipsoids drawn at the 30% probability level. H atoms are omitted for clarity. Symmetry codes: (A) 3/2-x, -1/2+y, z; (B) x, 1/2-y, 1/2+z; (C) 2-x, -y, 1-z.

**Fig. 2.**
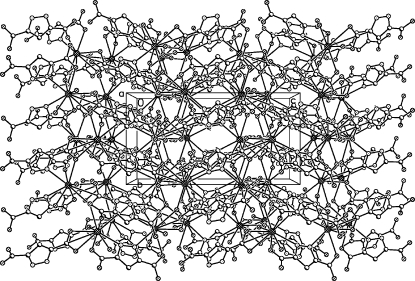
Crystal packing of the title compound viewed along the a axis. H atoms are omitted for clarity.

## supplementary crystallographic information

### Comment

In the past decade, the design and synthesis of metal–organic frameworks have drawn great attention. Pyrazole-3,5-dicarboxylic acid (H_3_pdc) has been found to be a suitable ligand in this study for its various coordination modes and strong coordination ability. Some complexes of H_3_pdc have been reported recently (Sakagami *et al.*, 1996; Wang *et al.*, 2007; Yang *et al.*, 2004; King *et al.*, 2003; Pan *et al.*, 2000). Herein, we report the synthesis and crystal structure of a new lanthanum complex with pdc^3-^ anions.

The coordination environment of the lanthanum(III) metal centre (Fig. 1) is provided by two N atoms and seven O atoms, of which the N atoms are from two pyrazole rings, five O atoms are from four carboxyl groups, and two O atoms are from water molecules. These atoms define a distorted tricapped trigonal prism, as commonly observed for nine-coordinate lanthanides. In the title complex, the La—O and La—N bond lengths are in the range 2.424 (2)–2.739 (2) and 2.621 (2)–2.665 (2) Å, respectively. The pdc^3-^ ligands link lanthanum(III) atoms to form a three-dimensional framework using a µ_4_ coordination mode (Fig. 2). The crystal structure is stabilized by O—H···O hydrogen bonds (Table 1).

### Experimental

All chemicals used (reagent grade) were commercially avaiable. The compound was synthesized by heating a mixture of pyrazole-3,5-dicarboxylic acid (0.078 g, 0.5 mmol), lanthanum nitrate (0.129 g, 0.3 mmol) and water (10 ml) in a 20 ml acid digestion bomb at 180 °C for 3 d. Colourless single crystals suitable for X-ray analysis were obtained after cooling to room temperature.

### Refinement

All H atoms were positioned geometrically and allowed to ride on their parent atoms, with C—H = 0.93, O—H = 0.85 Å and with *U*_iso_(H) = 1.2*U*_eq_(C) or 1.5*U*_eq_(O).

### Crystal data

**Table:**

[La(C_5_HN_2_O_4_)(H_2_O)_2_]	*F*(000) = 1232
*M**_r_* = 328.02	*D*_x_ = 2.567 Mg m^−^^3^
Orthorhombic, *P**b**c**a*	Mo *K*α radiation, λ = 0.71073 Å
Hall symbol: -P 2ac 2ab	Cell parameters from 3962 reflections
*a* = 12.5712 (8) Å	θ = 2.5–27.8°
*b* = 8.4350 (6) Å	µ = 5.04 mm^−^^1^
*c* = 16.007 (1) Å	*T* = 293 K
*V* = 1697.35 (19) Å^3^	Block, colourless
*Z* = 8	0.32 × 0.24 × 0.20 mm

### Data collection

**Table:**

Bruker APEXII CCD diffractometer	1496 independent reflections
Radiation source: fine-focus sealed tube	1316 reflections with *I* > 2σ(*I*)
graphite	*R*_int_ = 0.023
φ and ω scans	θ_max_ = 25.0°, θ_min_ = 3.0°
Absorption correction: multi-scan (*SADABS*; Sheldrick, 1996)	*h* = −14→13
*T*_min_ = 0.239, *T*_max_ = 0.365	*k* = −10→10
8554 measured reflections	*l* = −19→15

### Refinement

**Table:**

Refinement on *F*^2^	Secondary atom site location: difference Fourier map
Least-squares matrix: full	Hydrogen site location: inferred from neighbouring sites
*R*[*F*^2^ > 2σ(*F*^2^)] = 0.016	H-atom parameters constrained
*wR*(*F*^2^) = 0.041	*w* = 1/[σ^2^(*F*_o_^2^) + (0.0185*P*)^2^ + 2.3502*P*] where *P* = (*F*_o_^2^ + 2*F*_c_^2^)/3
*S* = 1.06	(Δ/σ)_max_ = 0.003
1496 reflections	Δρ_max_ = 0.52 e Å^−^^3^
128 parameters	Δρ_min_ = −0.50 e Å^−^^3^
0 restraints	Extinction correction: *SHELXL97* (Sheldrick, 2008), Fc^*^=kFc[1+0.001xFc^2^λ^3^/sin(2θ)]^-1/4^
Primary atom site location: structure-invariant direct methods	Extinction coefficient: 0.00271 (13)

### Special details

**Table:**

Geometry. All e.s.d.'s (except the e.s.d. in the dihedral angle between two l.s. planes) are estimated using the full covariance matrix. The cell e.s.d.'s are taken into account individually in the estimation of e.s.d.'s in distances, angles and torsion angles; correlations between e.s.d.'s in cell parameters are only used when they are defined by crystal symmetry. An approximate (isotropic) treatment of cell e.s.d.'s is used for estimating e.s.d.'s involving l.s. planes.
Refinement. Refinement of *F*^2^ against ALL reflections. The weighted *R*-factor *wR* and goodness of fit *S* are based on *F*^2^, conventional *R*-factors *R* are based on *F*, with *F* set to zero for negative *F*^2^. The threshold expression of *F*^2^ > σ(*F*^2^) is used only for calculating *R*-factors(gt) *etc*. and is not relevant to the choice of reflections for refinement. *R*-factors based on *F*^2^ are statistically about twice as large as those based on *F*, and *R*- factors based on ALL data will be even larger.

### Fractional atomic coordinates and isotropic or equivalent isotropic displacement parameters (Å^2^)

**Table:**

	*x*	*y*	*z*	*U*_iso_*/*U*_eq_	
La1	0.968426 (12)	0.009678 (17)	0.662201 (9)	0.01104 (9)	
O1	0.80247 (17)	0.1816 (2)	0.67873 (12)	0.0212 (5)	
O2	0.68279 (17)	0.3455 (3)	0.62560 (14)	0.0274 (5)	
O3	0.9060 (2)	0.5049 (2)	0.31256 (13)	0.0222 (5)	
O4	0.98606 (16)	0.2761 (2)	0.29004 (12)	0.0170 (4)	
O5	1.0936 (2)	0.2221 (3)	0.60343 (15)	0.0325 (6)	
H5A	1.0942	0.1442	0.6371	0.049*	
H5B	1.0973	0.3066	0.6322	0.049*	
O6	1.15184 (19)	−0.0151 (2)	0.73037 (16)	0.0338 (6)	
H6A	1.1780	−0.1047	0.7434	0.051*	
H6B	1.1851	0.0664	0.7475	0.051*	
N1	0.89277 (19)	0.1436 (3)	0.52362 (14)	0.0162 (5)	
N2	0.93750 (19)	0.1715 (3)	0.44839 (14)	0.0165 (5)	
C1	0.7644 (2)	0.2632 (3)	0.61947 (19)	0.0153 (6)	
C2	0.8238 (2)	0.2624 (3)	0.53938 (17)	0.0155 (6)	
C3	0.8237 (2)	0.3719 (3)	0.47460 (18)	0.0190 (7)	
H3	0.7841	0.4646	0.4698	0.023*	
C4	0.8967 (2)	0.3099 (3)	0.41903 (17)	0.0160 (6)	
C5	0.9322 (2)	0.3675 (3)	0.33671 (17)	0.0154 (6)	

### Atomic displacement parameters (Å^2^)

**Table:**

	*U*^11^	*U*^22^	*U*^33^	*U*^12^	*U*^13^	*U*^23^
La1	0.01164 (12)	0.01181 (12)	0.00967 (12)	0.00021 (6)	0.00036 (6)	−0.00053 (6)
O1	0.0230 (12)	0.0285 (11)	0.0122 (11)	0.0074 (9)	0.0021 (9)	0.0032 (9)
O2	0.0223 (12)	0.0311 (12)	0.0287 (13)	0.0141 (10)	0.0086 (10)	0.0075 (10)
O3	0.0358 (13)	0.0158 (11)	0.0150 (10)	0.0056 (9)	0.0055 (10)	0.0026 (8)
O4	0.0217 (11)	0.0158 (10)	0.0134 (11)	0.0024 (8)	0.0033 (8)	−0.0008 (8)
O5	0.0410 (15)	0.0214 (11)	0.0351 (13)	−0.0112 (11)	0.0097 (12)	−0.0019 (10)
O6	0.0253 (13)	0.0264 (12)	0.0496 (16)	−0.0030 (10)	−0.0187 (12)	0.0042 (10)
N1	0.0190 (13)	0.0176 (12)	0.0120 (12)	0.0019 (10)	0.0034 (10)	0.0015 (9)
N2	0.0201 (13)	0.0186 (12)	0.0108 (12)	0.0022 (10)	0.0041 (10)	0.0027 (10)
C1	0.0151 (16)	0.0149 (14)	0.0159 (15)	−0.0019 (12)	0.0012 (11)	−0.0016 (11)
C2	0.0159 (15)	0.0169 (14)	0.0137 (15)	0.0016 (12)	0.0000 (12)	0.0006 (11)
C3	0.0235 (17)	0.0163 (14)	0.0173 (16)	0.0060 (12)	0.0011 (12)	0.0011 (12)
C4	0.0190 (16)	0.0153 (13)	0.0137 (15)	0.0014 (12)	0.0004 (12)	0.0016 (11)
C5	0.0159 (15)	0.0153 (14)	0.0149 (16)	−0.0021 (11)	−0.0020 (11)	0.0005 (12)

### Geometric parameters (Å, °)

**Table:**

La1—O2^i^	2.424 (2)	O4—La1^iii^	2.5929 (19)
La1—O3^ii^	2.535 (2)	O4—La1^v^	2.7388 (19)
La1—O1	2.554 (2)	O5—H5A	0.8500
La1—O6	2.559 (2)	O5—H5B	0.8500
La1—O5	2.564 (2)	O6—H6A	0.8500
La1—O4^iii^	2.5929 (19)	O6—H6B	0.8499
La1—N2^iii^	2.620 (2)	N1—C2	1.349 (4)
La1—N1	2.665 (2)	N1—N2	1.350 (3)
La1—O4^ii^	2.7388 (19)	N2—C4	1.360 (4)
La1—C5^ii^	3.014 (3)	N2—La1^iii^	2.620 (2)
La1—H5A	1.9874	C1—C2	1.483 (4)
O1—C1	1.266 (4)	C2—C3	1.389 (4)
O2—C1	1.242 (3)	C3—C4	1.380 (4)
O2—La1^iv^	2.424 (2)	C3—H3	0.9300
O3—C5	1.266 (3)	C4—C5	1.474 (4)
O3—La1^v^	2.535 (2)	C5—La1^v^	3.014 (3)
O4—C5	1.269 (3)		
			
O2^i^—La1—O3^ii^	87.64 (7)	O2^i^—La1—H5A	154.3
O2^i^—La1—O1	73.08 (8)	O3^ii^—La1—H5A	117.8
O3^ii^—La1—O1	71.11 (7)	O1—La1—H5A	110.3
O2^i^—La1—O6	139.71 (7)	O6—La1—H5A	54.3
O3^ii^—La1—O6	82.55 (8)	O5—La1—H5A	15.9
O1—La1—O6	137.56 (7)	O4^iii^—La1—H5A	114.5
O2^i^—La1—O5	142.39 (7)	N2^iii^—La1—H5A	80.6
O3^ii^—La1—O5	124.93 (7)	N1—La1—H5A	82.7
O1—La1—O5	98.20 (7)	O4^ii^—La1—H5A	73.1
O6—La1—O5	70.16 (8)	C5^ii^—La1—H5A	96.4
O2^i^—La1—O4^iii^	73.32 (7)	C1—O1—La1	122.68 (18)
O3^ii^—La1—O4^iii^	75.12 (6)	C1—O2—La1^iv^	170.3 (2)
O1—La1—O4^iii^	132.65 (6)	C5—O3—La1^v^	99.48 (17)
O6—La1—O4^iii^	66.39 (6)	C5—O4—La1^iii^	120.58 (17)
O5—La1—O4^iii^	128.50 (7)	C5—O4—La1^v^	89.78 (16)
O2^i^—La1—N2^iii^	81.81 (8)	La1^iii^—O4—La1^v^	148.37 (8)
O3^ii^—La1—N2^iii^	138.85 (7)	La1—O5—H5B	114.9
O1—La1—N2^iii^	140.31 (7)	H5A—O5—H5B	107.7
O6—La1—N2^iii^	80.43 (8)	La1—O6—H6A	121.7
O5—La1—N2^iii^	83.25 (7)	La1—O6—H6B	120.9
O4^iii^—La1—N2^iii^	63.74 (7)	H6A—O6—H6B	116.7
O2^i^—La1—N1	76.18 (7)	C2—N1—N2	107.8 (2)
O3^ii^—La1—N1	134.47 (7)	C2—N1—La1	112.86 (17)
O1—La1—N1	63.52 (7)	N2—N1—La1	131.91 (17)
O6—La1—N1	135.32 (8)	N1—N2—C4	107.5 (2)
O5—La1—N1	67.51 (8)	N1—N2—La1^iii^	133.56 (17)
O4^iii^—La1—N1	135.91 (6)	C4—N2—La1^iii^	115.97 (17)
N2^iii^—La1—N1	81.13 (7)	O2—C1—O1	123.8 (3)
O2^i^—La1—O4^ii^	128.39 (7)	O2—C1—C2	119.1 (3)
O3^ii^—La1—O4^ii^	49.28 (6)	O1—C1—C2	117.0 (2)
O1—La1—O4^ii^	67.31 (6)	N1—C2—C3	110.8 (3)
O6—La1—O4^ii^	70.28 (7)	N1—C2—C1	119.2 (2)
O5—La1—O4^ii^	76.32 (6)	C3—C2—C1	129.9 (3)
O4^iii^—La1—O4^ii^	112.04 (2)	C4—C3—C2	103.2 (2)
N2^iii^—La1—O4^ii^	148.53 (7)	C4—C3—H3	128.4
N1—La1—O4^ii^	111.79 (6)	C2—C3—H3	128.4
O2^i^—La1—C5^ii^	107.57 (8)	N2—C4—C3	110.7 (2)
O3^ii^—La1—C5^ii^	24.47 (7)	N2—C4—C5	118.5 (2)
O1—La1—C5^ii^	65.50 (7)	C3—C4—C5	130.8 (3)
O6—La1—C5^ii^	76.66 (8)	O3—C5—O4	121.0 (3)
O5—La1—C5^ii^	101.11 (7)	O3—C5—C4	119.7 (3)
O4^iii^—La1—C5^ii^	94.58 (7)	O4—C5—C4	119.2 (2)
N2^iii^—La1—C5^ii^	153.48 (8)	O3—C5—La1^v^	56.04 (14)
N1—La1—C5^ii^	124.88 (7)	O4—C5—La1^v^	65.32 (14)
O4^ii^—La1—C5^ii^	24.90 (7)	C4—C5—La1^v^	171.1 (2)
			
O2^i^—La1—O1—C1	−89.3 (2)	La1—N1—N2—La1^iii^	55.4 (3)
O3^ii^—La1—O1—C1	177.3 (2)	La1—O1—C1—O2	178.7 (2)
O6—La1—O1—C1	122.6 (2)	La1—O1—C1—C2	−3.1 (3)
O5—La1—O1—C1	53.1 (2)	N2—N1—C2—C3	−0.9 (3)
O4^iii^—La1—O1—C1	−135.8 (2)	La1—N1—C2—C3	152.7 (2)
N2^iii^—La1—O1—C1	−36.2 (3)	N2—N1—C2—C1	−178.8 (2)
N1—La1—O1—C1	−6.6 (2)	La1—N1—C2—C1	−25.2 (3)
O4^ii^—La1—O1—C1	124.5 (2)	O2—C1—C2—N1	−161.5 (3)
C5^ii^—La1—O1—C1	151.7 (2)	O1—C1—C2—N1	20.3 (4)
O2^i^—La1—N1—C2	93.7 (2)	O2—C1—C2—C3	21.1 (5)
O3^ii^—La1—N1—C2	21.3 (2)	O1—C1—C2—C3	−157.2 (3)
O1—La1—N1—C2	15.99 (18)	N1—C2—C3—C4	0.4 (3)
O6—La1—N1—C2	−115.99 (19)	C1—C2—C3—C4	178.0 (3)
O5—La1—N1—C2	−96.26 (19)	N1—N2—C4—C3	−0.9 (3)
O4^iii^—La1—N1—C2	141.00 (17)	La1^iii^—N2—C4—C3	162.30 (19)
N2^iii^—La1—N1—C2	177.4 (2)	N1—N2—C4—C5	−179.6 (2)
O4^ii^—La1—N1—C2	−32.5 (2)	La1^iii^—N2—C4—C5	−16.4 (3)
C5^ii^—La1—N1—C2	−8.2 (2)	C2—C3—C4—N2	0.3 (3)
O2^i^—La1—N1—N2	−120.8 (2)	C2—C3—C4—C5	178.8 (3)
O3^ii^—La1—N1—N2	166.70 (19)	La1^v^—O3—C5—O4	7.1 (3)
O1—La1—N1—N2	161.4 (2)	La1^v^—O3—C5—C4	−170.7 (2)
O6—La1—N1—N2	29.5 (3)	La1^iii^—O4—C5—O3	−177.2 (2)
O5—La1—N1—N2	49.2 (2)	La1^v^—O4—C5—O3	−6.5 (3)
O4^iii^—La1—N1—N2	−73.6 (2)	La1^iii^—O4—C5—C4	0.7 (3)
N2^iii^—La1—N1—N2	−37.1 (2)	La1^v^—O4—C5—C4	171.4 (2)
O4^ii^—La1—N1—N2	113.0 (2)	La1^iii^—O4—C5—La1^v^	−170.65 (16)
C5^ii^—La1—N1—N2	137.2 (2)	N2—C4—C5—O3	−171.1 (3)
C2—N1—N2—C4	1.1 (3)	C3—C4—C5—O3	10.5 (5)
La1—N1—N2—C4	−145.6 (2)	N2—C4—C5—O4	11.0 (4)
C2—N1—N2—La1^iii^	−157.9 (2)	C3—C4—C5—O4	−167.4 (3)

Symmetry codes: (i) −*x*+3/2, *y*−1/2, *z*; (ii) *x*, −*y*+1/2, *z*+1/2; (iii) −*x*+2, −*y*, −*z*+1; (iv) −*x*+3/2, *y*+1/2, *z*; (v) *x*, −*y*+1/2, *z*−1/2.

### Hydrogen-bond geometry (Å, °)

**Table:**

*D*—H···*A*	*D*—H	H···*A*	*D*···*A*	*D*—H···*A*
O5—H5A···O6	0.85	2.14	2.944 (3)	159
O5—H5B···O3^vi^	0.85	1.82	2.667 (3)	174
O6—H6A···O1^vii^	0.85	2.20	2.999 (3)	155
O6—H6B···O1^viii^	0.85	2.12	2.908 (3)	153

Symmetry codes: (vi) −*x*+2, −*y*+1, −*z*+1; (vii) −*x*+2, *y*−1/2, −*z*+3/2; (viii) *x*+1/2, *y*, −*z*+3/2.
